# Status of New Vaccine Introduction — Worldwide, 2016–2021

**DOI:** 10.15585/mmwr.mm7227a2

**Published:** 2023-07-07

**Authors:** Gurpreet Kaur, Rebecca M. Casey, Jaymin C. Patel, Paul Bloem, Jenny A. Walldorf, Terri B. Hyde

**Affiliations:** ^1^Epidemic Intelligence Service, CDC; ^2^Global Immunization Division, Center for Global Health, CDC; ^3^Department of Immunization, Vaccines and Biologicals, World Health Organization, Geneva, Switzerland.

**Summary**

**What is already known about this topic?**

The global Immunization Agenda 2021–2030 (IA2030) aims to increase equitable access to and use of new and existing vaccines. The COVID-19 pandemic caused widespread disruption to routine immunization services.

**What is added by this report?**

By 2021, 17% of countries worldwide provided all eight World Health Organization–recommended new and underutilized vaccines in their routine immunization schedules. The number of new vaccines added to a national immunization program declined sharply at the start of the COVID-19 pandemic, from 48 in 2019 to 15 in 2020.

**What are the implications for public health practice?**

To achieve IA2030 targets, increased efforts to accelerate introductions of new and underutilized vaccines are urgently needed to facilitate equitable access, including to vaccines delivered beyond the first year of life.

## Abstract

This report describes the status of introductions globally for eight World Health Organization (WHO)–recommended new and underutilized vaccines, comprising 10 individual vaccine antigens. By 2021, among 194 countries worldwide, 33 (17%) provided all of these 10 WHO-recommended antigens as part of their routine immunization schedules; only one low-income country had introduced all of these recommended vaccines. Universal hepatitis B birth dose; human papillomavirus vaccine; rotavirus vaccine; and diphtheria, tetanus, and pertussis–containing vaccine first booster dose have been introduced by 57%, 59%, 60%, and 72% of all countries worldwide, respectively. Pneumococcal conjugate vaccine, rubella-containing vaccine, measles-containing vaccine second dose, and *Haemophilus influenzae* type b vaccine have been introduced by 78%, 89%, 94%, and 99% of all countries, respectively. The annual rate of new vaccine introductions declined precipitously when the COVID-19 pandemic started, from 48 in 2019 to 15 in 2020 before rising to 26 in 2021. Increased efforts to accelerate new and underutilized vaccine introductions are urgently needed to improve universal equitable access to all recommended vaccines to achieve the global Immunization Agenda 2021–2030 (IA2030) targets.

## Introduction

The global Immunization Agenda 2021–2030 (IA2030), by increasing equitable access to and use of new and existing vaccines, envisions a world where everyone everywhere fully benefits from vaccines. IA2030, endorsed by the World Health Assembly, includes a target to achieve 500 new and underutilized vaccine introductions in low-income and middle-income countries’ routine immunization schedules by 2030 (*1*). This report updates a 2016 report (*2*) and describes the status of introductions globally for eight World Health Organization (WHO)–recommended new and underutilized vaccines, comprising 10 individual vaccine antigens, including five provided beyond the first year of life.

## Methods

Data on the status of national introductions of eight WHO-recommended new and underutilized vaccines in 194 countries were obtained from the WHO/UNICEF Immunization database (*3*); five of these vaccines are provided during infancy (hepatitis B birth dose [HepB-BD], *Haemophilus influenzae* type b vaccine [Hib], pneumococcal conjugate vaccine [PCV], rubella-containing vaccine [RCV], and rotavirus [RV] vaccine), and three vaccines including a total of five antigens are provided after the first year of life (fourth diphtheria, tetanus, and pertussis-containing vaccine [first booster] dose [DTPCV4],* human papillomavirus vaccine [HPV], and measles-containing vaccine second dose [MCV2]) (*4*). The number of new vaccine introductions globally was calculated for each year during 2016–2021 and compared with average number of annual new vaccine introductions during 2010–2015. Vaccine introduction status is additionally reported according to country income category as defined by the World Bank^†^ (*5*).

## Results

### New and Underutilized Vaccines Included in National Schedules by 2021

By the end of 2021, among 194 countries, universal HepB-BD was included in the national immunization schedules in 111 (57%) countries, RV in 116 (60%), PCV in 152 (78%), and RCV in 173 (89%). HepB-BD, targeting only select populations, was included in the routine immunization schedules of 24 (12%) countries, mostly in the WHO European Region (EUR) (20 of 24 countries). Hib vaccine was included in the national immunization schedules of all but two (99%) countries (China and Russia). Beyond the first year of life, HPV vaccine, DTPCV4, and MCV2 were included in the national immunization schedules of 114 (59%), 140 (72%), and 183 (94%) countries, respectively.

### New and Underutilized Vaccine Introductions, 2016–2021

During 2016–2021, among vaccines recommended during the first year of life, the numbers and percentage of countries that had implemented national vaccine introduction increased most for RV vaccine, from 84 (43%) countries to 116 (60%) (Table) (Supplementary Figure, https://stacks.cdc.gov/view/cdc/130137). During 2016–2021, among vaccines provided during the second year of life and beyond, the number of new HPV vaccine introductions was higher than the number of introductions of any other recommended vaccine; the number of countries that had introduced HPV vaccine nationally increased 65%, from 69 countries in 2016 to 114 in 2021. During the same period, the number of countries that had introduced MCV2 increased by 12%, from 164 countries in 2016 to 183 in 2021; countries introducing DTPCV4 increased by 4%, from 135 countries in 2016 to 140 in 2021. By 2021, 33 (17%) of 194 countries provided all eight of these WHO-recommended new and underutilized vaccines as part of the routine immunization schedule; 56 (29%) countries had included all five vaccines recommended during the first year of life.

**TABLE:** Number and percentage of countries that included eight World Health Organization–recommended new and underutilized vaccines* available in their national routine immunization schedule, by year — worldwide, 2016–2021

Year	No. (%), yr WHO recommended†							
	DTPCV4 2017^§^	HepB-BD 2009	Hib 2006	HPV 2009^¶^	MCV2 2009	PCV 2007	RCV 2000**	RV 2009
2016	135 (70)	100 (52)	190 (98)	69 (36)	164 (85)	132 (68)	155 (80)	84 (43)
2017	136 (70)	104 (54)	190 (98)	79 (41)	167 (86)	135 (70)	160 (82)	91 (47)
2018	137 (71)	106 (55)	191 (98)	87 (45)	171 (88)	138 (71)	168 (87)	95 (49)
2019	138 (71)	109 (56)	192 (99)	103 (53)	177 (91)	144 (74)	173 (89)	105 (54)
2020	137 (71)	110 (57)	192 (99)	107 (55)	179 (92)	146 (75)	173 (89)	111 (57)
2021	140 (72)	111 (57)	192 (99)	114 (59)	183 (94)	152 (78)	173 (89)	116 (60)

**Abbreviations**: DTPCV4 = first booster dose of diphtheria, tetanus, and pertussis–containing vaccine; HepB-BD = universal hepatitis B vaccine birth dose; Hib = *Haemophilus influenzae* type b vaccine; HPV = human papillomavirus vaccine; MCV2 = second dose of measles-containing vaccine; PCV = pneumococcal conjugate vaccine; RCV = rubella-containing vaccine; RV = rotavirus vaccine; WHO = World Health Organization.

* Vaccine introduction data for DTPCV4 was unavailable for 2016. For all other vaccines, no value indicates no introductions occurred for that year.

† Year WHO recommended inclusion of vaccine in all national routine immunization programs.

**^§^** In 2017, WHO revised its DTPCV booster recommendations, shifting the first booster dose of tetanus to the second year of life to align with the recommendation for the first booster dose of pertussis. Countries reporting inclusion of DTPCV4 in this table might provide it at any age.

**^¶^** HPV was originally recommended as a 3-dose schedule for girls aged 9–13 years in 2009 and updated to a 2-dose schedule recommendation in 2014 for girls aged 9–14 years; an alternative single-dose schedule was recommended in 2022 for girls aged 9–14 years.

** In 2000, WHO recommended introduction of RCV in countries where it can be safely introduced.

**FIGURE 1:**
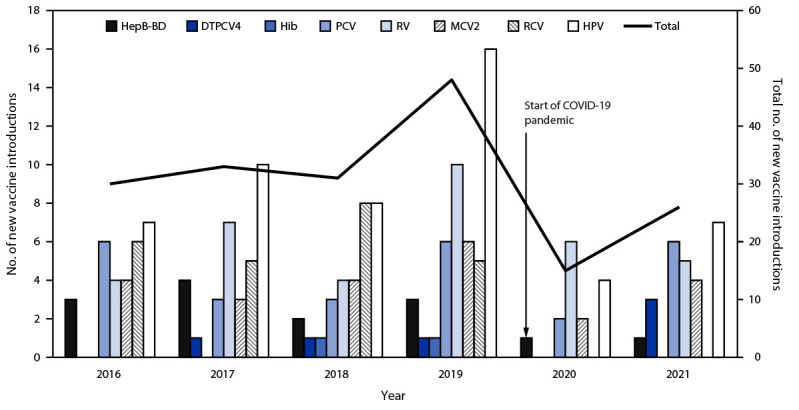
Number of countries with new vaccine introductions, by vaccine and year — worldwide,* 2016–2021 **Abbreviations:** DTPCV4 = first booster dose of diphtheria, tetanus, and pertussis–containing vaccine; HepB-BD = hepatitis B vaccine birth dose; Hib = Haemophilus influenzae type b vaccine; HPV = human papillomavirus vaccine; MCV2 = second dose of measles-containing vaccine; PCV = pneumococcal conjugate vaccine; RCV = rubella-containing vaccine; RV = rotavirus vaccine. * Vaccine introduction data for DTPCV4 was unavailable for 2016. For all other vaccines, no value indicates no introductions occurred for that year.

During 2016–2021, an average of 31 new introductions of these eight WHO-recommended new and underutilized vaccines occurred annually (representing a decline in introductions of approximately one third [34%]), compared with an average 47 new introductions annually during 2010–2015.^§^ The annual number of new vaccine introductions declined sharply at the start of the COVID-19 pandemic, from 48 in 2019 to 15 in 2020 (Figure 1). In 2021, there were 26 vaccine introductions, including one HepB-BD, three DTPCV4, six PCV, five RV, four MCV2, and seven HPV introductions.

### New and Underutilized Vaccine Introductions, by World Bank Income Status and WHO Region

**FIGURE 2:**
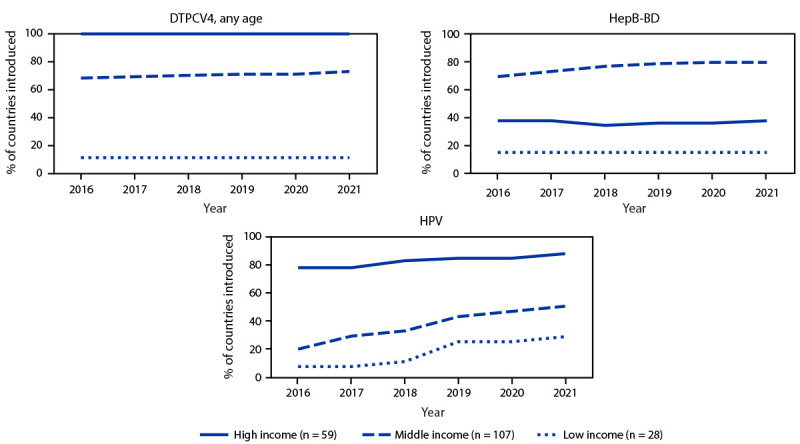
Percentage of countries that introduced selected World Health Organization–recommended vaccines* into their national immunization schedule, by income status^†^ — worldwide, 2016–2021 **Abbreviations:** DTPCV4 = first booster dose of diphtheria, tetanus, and pertussis–containing vaccine; HepB-BD = hepatitis B vaccine birth dose; HPV = human papillomavirus vaccine; USD = U.S. dollars. * Vaccines with lowest introduction in low-income countries' national immunization schedules as of 2021. HepB-BD indicates the introduction of universal HepB-BD into the national immunization schedule. In addition, 24 countries had implemented HepB-BD selective introduction, of which 22 were high-income and two were middle-income countries. ^†^ Country income categories were defined using the 2022 World Bank income classification except for Cook Islands, Niue, and Venezuela. Cook Islands is classified as high-income and Niue is classified as middle-income based on Central Intelligence Agency classification (https://www.cia.gov/the-world-factbook/countries/). Venezuela is classified as middle-income based on that country’s most recent World Bank classification (2019). Gross national income: low income <1,085 USD; middle income = 1,086–13,205 USD; and high income >13,205 USD.

By 2021, among 28 low-income countries, 20 (71%) had yet to introduce HPV vaccine into their routine immunization schedule compared with 53 (50%) of 107 middle-income countries and seven (12%) of 59 high-income countries (Figure 2). RV vaccine had not yet been introduced in 25%, 39%, and 49% of low-, middle- and high-income countries, respectively. As well, minimal progress toward DTPCV4 and HepB-BD vaccine introductions was made across all country income categories during 2016–2021.

Among the 152 vaccine introductions implemented during 2016–2021, 58 (38%) were in the African Region (AFR); 28 (18%) in the Region of the Americas (AMR); 23 (15%) in the Western Pacific Region (WPR); 21 (14%) in EUR; 17 (11%) in the South-East Asia Region (SEAR); and five (3%) in the Eastern Mediterranean Region (EMR). As of 2021, by vaccine, the largest proportion of countries that had not introduced HepB-BD (72%), DTPCV4 (79%), MCV2 (19%), and RCV (34%) were in AFR. Among 21 countries in EMR, 19 (90%) had yet to introduce HPV vaccine. By schedule, an average of 29% of countries in AFR had yet to introduce one or more of five WHO-recommended infant vaccines followed by 27% in SEAR, 25% in EUR, 22% in EMR, 20% in AMR, and 14% in WPR. Beyond the infant schedule, an average of 51% countries in AFR had yet to introduce at least one of HPV, DTPCV4, or MCV2 into the national immunization schedule, followed by 40% in EMR, 30% in SEAR, 23% in WPR, 9% in EUR, and 4% in AMR.

## Discussion

Worldwide, the introduction of WHO-recommended vaccines into national immunization schedules has increased overall since 2016; however, the rate of new vaccine introductions slowed during the COVID-19 pandemic. More than two thirds of the 194 WHO member countries have now individually introduced Hib vaccine (99%), MCV2 (94%), RCV (89%), PCV (78%), and DTPCV4 (72%). In low-income countries, DTPCV4, HepB-BD, and HPV vaccine are the most underutilized vaccines, whereas in high-income countries, RV vaccine is the most underutilized vaccine. Enhanced efforts are needed to revitalize national vaccine introductions and prioritize equitable access to all vaccines, including those provided beyond infancy, through adolescence, and across the life-course.

A reliable supply of affordable vaccines is required to facilitate new vaccine introductions and achieve high vaccination coverage to achieve the goals outlined in the IA2030, which include achieving at least 500 cumulative vaccine introductions in low- and middle-income countries by 2030; as of 2021, 167 cumulative vaccine introductions had been achieved (*1*). During the last 5 years, availability of RV and HPV vaccines has suffered from supply constraints, which have affected vaccine introductions; these constraints have been exacerbated by the COVID-19 pandemic (*4*,*6*). Global partners including Gavi, the Vaccine Alliance, have been working to address challenges around supply and affordability so that the growing portfolio of newly licensed vaccines can be introduced worldwide more rapidly.

In 2016, Gavi began providing support for the national scale-up of HPV vaccine introductions in low- and middle-income countries after small-scale subnational demonstration projects during 2011–2015 determined that introduction was feasible. Despite vaccine supply challenges, the percentage of countries that had introduced HPV vaccine into national programs during 2016–2021 increased by 65%, which is more than the global increase in introductions for any other vaccine. Fifty-seven low- and middle-income countries qualified for Gavi support during 2016–2021, but this support was limited to low- and lower-middle-income countries. Among middle-income countries, which can be further subdivided into lower-middle-income and upper-middle-income, no upper-middle-income countries qualified for Gavi support during 2016–2021. As a result of the COVID-19 pandemic, Gavi paused its investment in certain vaccines (such as DTPCV4 and HepB-BD), which hindered progress for those vaccines introductions (*7*,*8*).

The vaccine access challenges in middle-income countries and the need for increased action have been subjects of increasing focus. Middle-income countries still report that the cost of vaccines is a major obstacle to their introduction, in part because of a lack of procurement capacity and suboptimal in-country regulatory processes (*1*). In December 2020, Gavi approved a new approach to engaging with middle-income countries that were formerly Gavi-eligible and selected countries that have never been Gavi-eligible to drive the sustainable introduction of important vaccines that have not yet been introduced, including HPV and RV vaccines. In addition, in 2022, WHO recommended that HPV vaccination may optionally be delivered as a single dose vaccine (*4*), which will likely increase programmatic feasibility and flexibility and reduce cost. These efforts to improve targeted donor funding and timely evidence-based updates to global vaccination policy are critical to ensuring continued progress in successful new vaccine introductions worldwide.

The delayed introduction of RV vaccine in high-income countries suggests that challenges beyond vaccine cost or interrupted supply, such as the lack of awareness among policymakers about the benefits of RV vaccine against the impact of rotavirus-related disease, and the lasting negative impact of safety concerns (*9*) are affecting introductions. Given the evidence that the benefits of RV vaccine far exceed the risks in low-, middle-, and high-income countries (*9*), high-level advocacy is needed globally to encourage the prioritization of RV vaccine and other underutilized vaccines. Additions to the evidence base supporting safety and effectiveness of these vaccines in specific contexts can further drive demand and support for these vaccine introductions into country routine immunization schedules. To accelerate global access to DTPCV4 and HepB-BD, targeted funding to support introductions, innovative strategies to address country awareness, and logistical support (e.g., trained staff members and cold chain management) to ensure timely access to vaccination, are needed (*9**,**10*).

### Limitations

The findings in this report are subject to at least three limitations. First, routine immunization schedule data reported by countries to WHO and UNICEF might not reflect national availability of vaccine. Second, incomplete data reported for some vaccines (e.g., DTPCV4 for years 2012, 2013, 2015, and 2016) limited the accuracy of the annual number of vaccine introductions for these years. Finally, World Bank income classification was not available for all countries, so alternative data sources or alternative years were used in calculations. 

### Implications for Public Health Practice

It is encouraging that, despite the disruptions to essential health services during the COVID-19 pandemic, many countries continued to introduce vaccines into their national schedules during 2020–2021. However, the COVID-19 pandemic slowed progress, and urgent recovery actions are needed. The COVID-19 vaccination response during the pandemic has highlighted the importance of building strong vaccination delivery platforms through childhood and across the life-course. To achieve IA2030 targets, increased efforts to accelerate introductions of new and underutilized vaccines are urgently needed to facilitate equitable access, including access to vaccines delivered beyond the first year of life.

## Footnotes

* DTPCV4 refers to the fourth dose of diphtheria, tetanus, and pertussis–containing vaccine administered at any age. DTPCV4 is one vaccine composed of three antigens.

† Country income categories were defined using the 2022 World Bank income classification except for Cook Islands, Niue, and Venezuela. Cook Islands is classified as high-income, and Niue is classified as middle-income based on Central Intelligence Agency classification (https://www.cia.gov/the-world-factbook/countries). Venezuela is classified as middle-income based on that country’s most recent World Bank classification (2019).

§ Introduction data for DTPCV4 are not available in average calculation for 2012, 2013, 2015, and 2016.
